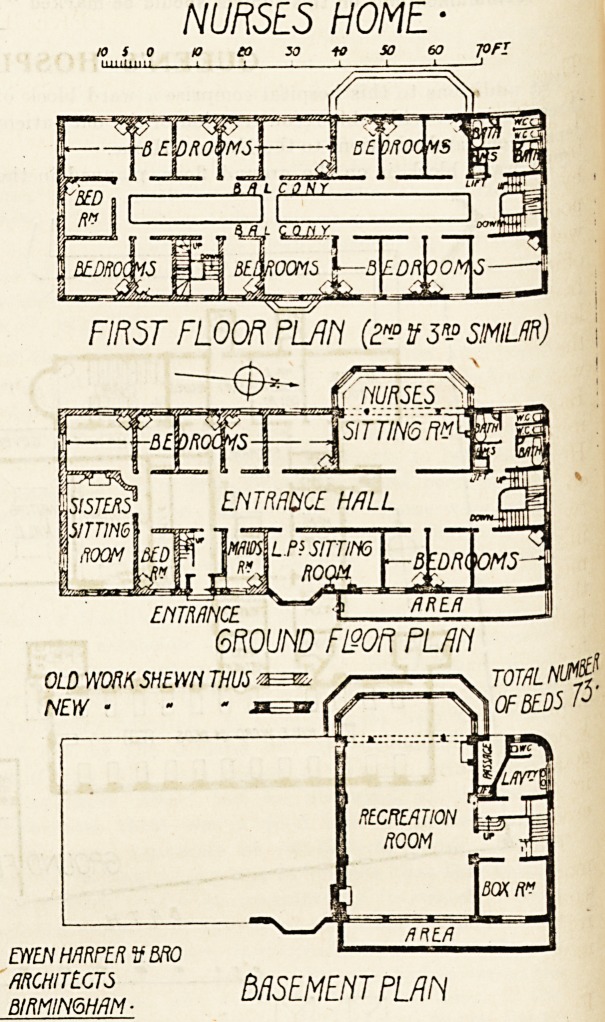# Queen's Hospital, Birmingham

**Published:** 1911-08-12

**Authors:** 


### Hospital Architecture and Construction.

[Communications on this subject should be marked "Architecture" in the left-hand top corner of the envelope.]

### QUEEN'S HOSPITAL, BIRMINGHAM.

The additions to this hospital comprise a ward block of three stories, considerable reconstruction of the out-patient department, and additions to the Nurses' Home.

The ward block is on the ground floor, planned in the form of an H; on the front or south side is a ward for twenty-four beds, with the sanitary offices projected out in wings on the north side. At right angles to the ward the connecting building contains the ward offices (kitchen, stores for clothes and linen, clinical room), also a room for electrical treatment, a day room for men, the entrance hall and staircase, and a cloak-room and lavatory for lady students. In the well of the staircase is a passenger lift.

It is unfortunate that a large space on the north side of the ward is blocked for ventilation purposes by the entrance and the ward offices, but in view of the restricted form of the site it is difficult to see how this could have been arranged otherwise.

The north wing contains a lecture-room, rooms for male students, staff and records, and chapel.

On the first floor the building takes the form of a T, the north wing being a ward for twenty-four beds, similar to the one below; while the wing running north and south contains, in addition to the ward offices and day room, four small wards for special cases. No sanitary offices are provided for these wards, so that all bed-pans would have to be carried along the corridor and through the large ward to the sink-room in the sanitary annexe.

On the second floor is a children's ward for fifteen cots and a male ward for nine beds.

All the cases treated in this new building will be medical, the old hospital being now devoted to surgical cases only.

The bath-rooms appear from the plan to be inconveniently small, and the space not sufficient to allow access to both sides of the bath, and it looks as if the carrying a patient into either of the bath-rooms would be a difficult, if not impossible, task; we are glad to note the provision of a w.c. for nurses entered out of the sink-room, an arrangement too often overlooked. The sink-room itself would have been better if double the size.

The alterations to the out-patient department consists largely of remodelling the existing building, which was built many years ago, and although then a great advance on anything that had gone before, had become quite inadequate for modern needs. A new dispensary and waiting-room for medicine, with a block of sanitary offices for patients, have been built out to the west of the old building, and the space thus set free has been utilised for the much-needed provision of additional consulting and examination rooms. Further accommodation has also been provided on the east side by building a one-storey addition. The additions have been skilfully planned, and the department is now as complete as it could be made in the circumstances.

The Nurses' Home would appear to have been quite inadequate for the needs of the hospital before the recent additions, as one-third of the nurses had to sleep out. The Home has now been enlarged, so that the whole of the nursing staff is housed, in addition to which a large recreation-room and two sitting-rooms have been provided, besides additional sanitary offices, box-room, etc.

The whole of these works have been planned and carried out by Messrs. Ewen Harper and Brother, of Birmingham.

## Figures and Tables

**Figure f1:**
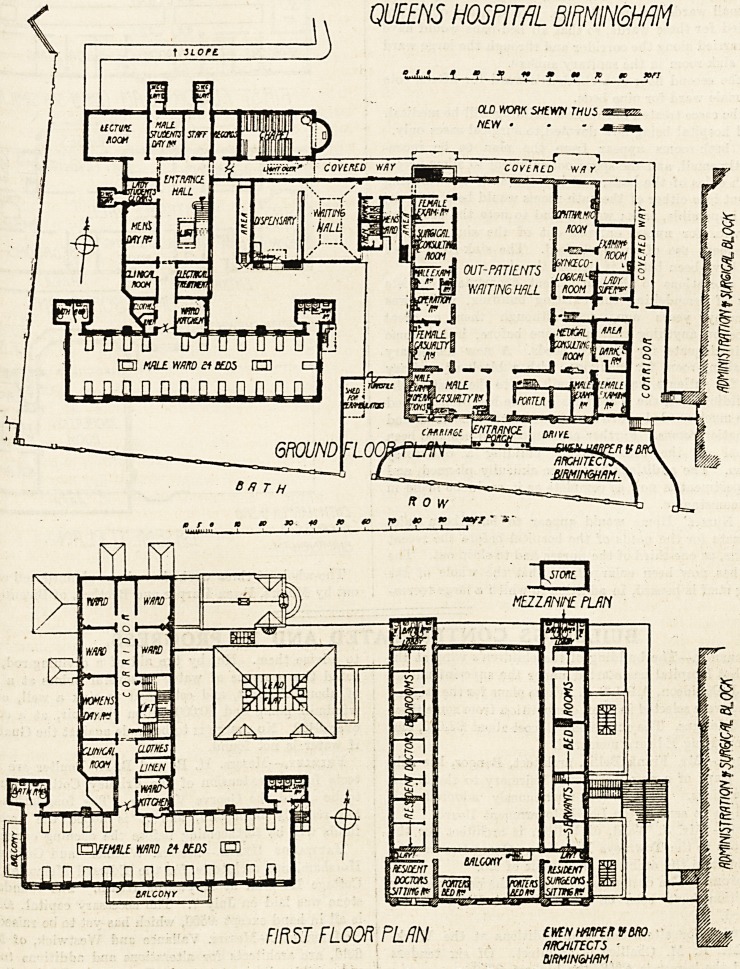


**Figure f2:**